# Challenges and constraints to the sustainability of poultry farming in China

**DOI:** 10.5713/ab.24.0794

**Published:** 2025-02-25

**Authors:** Fei Zhang, Zhuting Chen, Jiayi Shi, Chenglong Han, Qinyi Zhan, Zhouzheng Ren, Xiaojun Yang

**Affiliations:** 1 College of Animal Science and Technology, Northwest A&F University, Yangling, Shaanxi, China

**Keywords:** Digestive System, Genetic Resources, Intensive Husbandry, Poultry Feeding, Poultry Industry

## Abstract

China’s poultry industry is characterized by large-scale production and rich breeds, presenting both opportunities and challenges. In 2023, the industry produced 10.79 billion broilers, 28.38 million tons of eggs, and 4.88 billion waterfowl. The foundation of a thriving poultry industry lies in the continuous improvement of breeds. For instance, new lines of Lueyang black-boned chickens have been developed using genomic selection breeding, with a focus on improving production performance and unlocking their high-quality genetic potential. Precision nutrition programs enhance the expression of poultry’s genetic potential and improve feed utilization efficiency. The five-dimensional feed evaluation system and the comprehensive National Feed Database provide formulators with accurate nutritional parameters of feed. Additionally, the concept of “nutrition power” and the “five-ring gold standard” enable researchers to analyze poultry’s digestive physiology more effectively. Feeding management plays a crucial role in optimizing genetic potential and the effectiveness of precision nutrition. To further boost production efficiency, smart farming systems have been implemented, incorporating intelligent management of environmental factors, animal parameters, and poultry health tracking. Meanwhile, in order to improve material utilization efficiency across the entire poultry production chain and support the sustainable development of the poultry industry, it is essential to optimize and promote the application of the Poultry-Crop interaction systems. In summary, strengthening fundamental research in poultry, optimizing smart poultry farm platforms, and implementing Poultry-Crop Interacting systems will drive the sustainable development of China’s poultry industry.

## POULTRY PRODUCTION IN CHINA

### Poultry industry scale

In 2023, China poultry industry produced 7.195 billion white-feather broilers (equivalent to 4.673 million tons of meat), raised 1.189 billion laying hens (providing 28.39 million tons of eggs), as well as 4.218 billion meat ducks, 149 million laying ducks, and 515 million geese [1].

### Poultry products consumption structure

The diversity of poultry breeds offers consumers a wide range of cooking options. White-feather broilers, known for their affordability and tender meat, have become the dominant choice for public consumption. The muscle is commonly used in fried chicken and low-fat meals. However, their short feeding cycle results in a lack of rich flavor in the meat. In contrast, the longer rearing time of yellow-feather broilers provide tastier and more nutritious meat, making them ideal for dishes such as chicken soup. Additionally, ducks and geese, with their distinctive flavors, are prized ingredients for roast duck, stews, and pot-stewed dishes.

### Opportunity and challenge

1) Local poultry breeds are valuable resources for breeding new varieties with unique traits and optimizing those breeds with high productivity, but many of these resources remain underutilized and unexplored.
2) Precision nutrition programs for poultry optimize their genetic potential and improve feed efficiency. However, it remains essential to accurately detect the nutritional value of feed and thoroughly study the digestive physiological characteristics of poultry.
3) Smart poultry farming improves production efficiency across the entire poultry production chain. However, the effectiveness of smart poultry farm management systems and the parameters used to reflect animal health status require further research and optimization.
4) Poultry-Crop Interacting systems are vital for the sustainable development of the poultry industry. However, methods to effectively integrate poultry farming with crop cultivation and enhance the efficiency of these systems still require further study.

## INDIGENOUS POULTRY GENETIC RESOURCES

Environmental factors and human selection have shaped a diverse range of poultry genetic resources in China. According to the catalog of the National Genetic Resources Commission of China by 2020, local poultry breeds include 114 chicken breeds, 37 duck breeds, 30 goose breeds and 2 pigeon breeds [2]. Besides, several high-performance breeds were bred, including 81 chicken breeds, 8 duck breeds, 3 goose breeds, 1 pigeon breed, and 1 quail breed [2].

### Genetic resources of indigenous poultry

Host gene information and microbiome information jointly regulate the production phenotype of poultry. The first step in unlocking the potential of these genetic resources is constructing genome catalogs and microbiome catalog for China’s poultry breeds.

#### Establishment of a genome database in China

By integrating genome data from 15 globally representative poultry breeds, Chinese scholars built the first high-quality pan-genome assembly for poultry. This work identified genes related to immune pathways located in chromatin subtelomeric regions and microchromosomes, shedding light on poultry evolution [3]. Furthermore, in 2023, researchers identified 10 chicken microchromosomes with high guanine-cytosine content and highly methylated tandem repeats, resembling the W chromosome, highlighting unique genetic adaptations [4]. Pedigree analysis also reveals genetic connections among Chinese poultry breeds. For instance, an analysis of five gamecock breeds and nine indigenous chicken breeds demonstrated that southern Chinese domestic chickens played a significant role in the domestication of gamecocks [5]. Gene expression in poultry is regulated by numerous unknown transcription factors. By analyzing 377 whole-genome sequencing datasets from 23 adult chicken tissues, researchers predicted approximately 1.2 million enhancer gene pairs and 7,662 super-enhancers, providing a valuable resource for future research [6].

#### Establishment of a microbiome database in China

The combination of host genetics and microorganisms significantly impacts poultry performance. For example, a study analyzing genome, microbial composition, and residual feed intake in 206 male yellow-feather dwarf broilers found that host genetics contributed 39%, while intestinal microorganisms in different regions had varying effects: duodenum (14%), cecum (28%), feces (10%), and jejunum and ileum (0%) [7]. Similarly, in female Rhode Island Red chickens, feed conversion efficiency was influenced by genetics (28%) and the intestinal microbiome, with regional contributions from the jejunum (3%), ileum (15%), feces (10%), and others (duodenum and cecum) showing no effect. Residual feed intake was influenced by both genetics (48%) and gut microbes, with contributions from the jejunum (20%), cecum (11%), feces (10%), and other regions (duodenum and ileum) [8]. These findings suggest that host genetics may influence poultry performance through the gut microbiome. The genomic catalog of intestinal microbes provides basic information for analyzing the mechanism of microbial effect on poultry production performance. Advances in gut microbiome research have further enriched the field. A comprehensive chicken gut microbiome catalog has been established, including 18,201 bacterial genomes, 225 archaeal metagenome-assembled genomes, and 33,411 viral genomes. Researchers also identified 812 hypothetical species and 240 hypothetical bacterial genera, providing foundational data for analyzing microbial effects on poultry production performance [9].

In summary, significant progress has been made in building rich genome and microbiome databases. In the future, it will be essential to continuously expand the genome and microbiome databases, while identifying potential targets to further enhance poultry production performance.

### High production performance breeds

#### Representative breed

Guangming white-feather broiler, yellow-feathered broilers, Jinghong laying layers and Zhongxing ducks.

High production performance is a crucial economic indicator in poultry farming. High-performing poultry breeds have been developed by identifying and selecting key gene loci. Many studies continue to explore critical targets for regulating production performance. To investigate the mechanisms underlying high meat production, a study compared the genomes of high-performance chickens and local breeds. The analysis identified 83 differentially expressed genes, primarily associated with muscle development, with the myosin heavy polypeptide 1 (MHY1) gene being the most representative [10]. However, high growth performance in white-feather broilers is often accompanied by excessive abdominal fat deposition, which limits improvements in feed efficiency. By integrating genomics, epigenomics, 3D genomics, and transcriptomics, researchers constructed a comprehensive gene regulatory network. This revealed that the rs734209466 variant promotes the transcription of insulin like growth factor binding protein (IGFBP) 2 and IGFBP5 by binding to the transcription factor interferon regulatory factor 4 (IRF4), thereby influencing preadipocyte proliferation and differentiation [11]. Muscle lipid content is another key factor, significantly affecting the taste and nutritional value of meat. Using Jingxin yellow-feather broilers as breeding material, researchers bred a high intramuscular fat line to enhance meat quality [12]. Similarly, laying rate is a critical economic trait in poultry. By integrating multi-omics data from various tissues of Hy-Line Brown laying hens with differing laying rates, several candidate genes were identified. These include tissue factor pathway inhibitor 2 (TFPI2) (promoting gonadotropin-releasing hormone secretion in hypothalamic neurons), follicle stimulating hormone beta (FSHβ) and luteinizing hormone beta (LHβ) (stimulating pituitary cell secretion), osteocrin (OSTN) (enhancing granulosa cell proliferation and steroid hormone synthesis), apolipoprotein A4 (APOA4) in the liver, and angiopoietin like 2 (ANGPTL2) in adipose tissue [13].

### Rare breeds

Endemic rare poultry breeds are characterized by strong environmental adaptability, disease resistance, unique appearance, and high nutritional and flavor value. Among these, environmental adaptability is a trait often lacking in high-production poultry breeds. Genome-wide resequencing of four breeds from various temperature zones in China (Wenchang chicken, green-shell chicken, Tibetan chicken, and Lindian chicken) revealed genes associated with tropical (solute carrier family 1 and thyroid stimulating hormone receptor) and cold adaptation (ubiquinone oxidoreductase subunit S4) [14]. Additionally, studies of poultry from extreme environments in Xinjiang identified multiple temperature-tolerance genes and pathways associated with humidity tolerance (lysosome, cysteine and methionine metabolism, glycosaminoglycan degradation, and Wnt signaling) [15]. Adaptations such as curly feathers in southern chicken breeds, like the Kirin chicken, were linked to the keratin type II cytoskeletal 75-like 4 (KRT75L4) gene, facilitating survival in hot climates [16]. Rare breeds also exhibit unique disease resistance traits, as shown by the distinct major histocompatibility complex B locus in pheasants, which may provide material for breeding disease-resistant chickens [17]. These identified targets that enhance poultry’s environmental adaptability can be utilized to optimize high-production poultry breeds.

**Figure 1:**
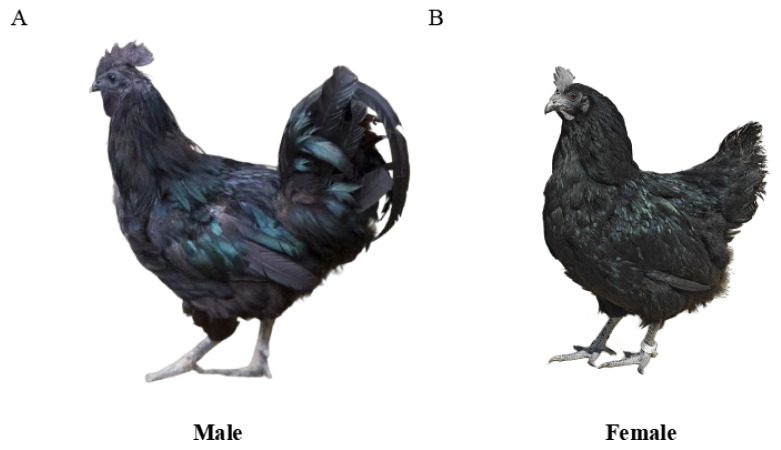
Photograph of Lueyang black-boned chicken. (A) Male Lueyang black-boned chicken. (B) Female Lueyang black-boned chicken.

In the case of the rare Lueyang black-boned chicken (Figure 1), our research team has enhanced its production performance through systematic breeding and is currently developing strains with high disease resistance. Lueyang black-boned chickens, originating in the Qinling Mountains (dating back to 25–200 AD), are prized for their high-quality meat and eggs. Genetic studies revealed high expression of kinase insert domain receptor (KIT), application specific instruction set processor (ASIP), tyrosinase (TYR), and oculocutaneous albinism type II (OCA2) genes in black-skin variants [18]. Furthermore, the unique blue-greenish eggshell color of Lueyang black-boned chickens was linked to the high frequency of haplotype 4 in the solute carrier organic anion transporter family member 1B3 (SLCO1B3) gene, rather than conventional retroviral insertions [19]. However, the production efficiency of Lueyang black-boned chickens is significantly lower than high-performance breed. In order to promote the development of Lueyang black-boned chicken industry, our research team collaborated with the local government to set up the Shaanxi Engineering and Technology Research Center of Lueyang black-boned chicken. This institute focuses on seven areas: breeding, precision nutrition, feeding management, disease prevention, new product development, functional poultry products, and industrial-scale enhancement. Key achievements include:

#### Breeding

Three specialized lines were bred: a meat line (At 10 weeks of age, male body weight increased about 300 g; female body weight increased about 120 g), an egg-laying line (To improve egg production rate and egg quality), and a disease-resistant line (enhanced resistance to salmonella by reducing the heterophil-to-lymphocyte ratio) [20].

#### Precision nutrition

A precise nutrition standard tailored to the age and physiological state of Lueyang black-boned chickens was established, significantly enhancing their production efficiency.

#### Feeding management

Based on the natural living habits of Lueyang black-boned chickens in mountainous regions, a breeding system was adopted that involves cage rearing during the brooding period and free-range rearing during the growing period. This approach respects the animals’ natural behaviors while improving growth efficiency.

#### Disease control

Based on the disease challenges faced by Lueyang black-boned chickens, the mortality rate was significantly reduced through the implementation of an individualized standard plan, which included provenance purification, tailored vaccination schedules, and strict feeding management.

#### Product development

Collaboration with food scientists to create new products that leverage the unique flavor and nutritional value of Lueyang black-boned chicken.

#### Functional poultry products

Incorporation of high-quality Chinese herbs from the Qinling Mountains into feed to produce functional poultry products.

#### Regional economic impact

Over more than a decade of systematic breeding of Lueyang black-boned chickens, coupled with the formulation of specialized feeding management programs and the development of distinctive poultry products, the breeding population has grown from 2.03 million birds in 2010 to 2.95 million birds in 2024 [21,22].

## FEED EFFICIENCY

According to the Food and Agriculture Organization of the United Nations (FAO), global food production is projected to reach approximately 2.8 billion tons by 2024. Among them, wheat, rice and maize are the main food crops, yielding about 770 million tons, 550 million tons and 1.16 billion tons respectively [23]. However, the world is facing challenges such as climate change, soil degradation and water scarcity, which affect the sustainability of food production. Among them, China’s total grain output is 665 million tons, of which the output of rice, corn and wheat is 210 million tons, 260 million tons and 130 million tons respectively. In 2023, the total output of grain used for animal feed in China will be about 321.627 million tons, of which 298.885 million tons of complete formula, 14.188 million tons of concentrate, and 7.091 million tons of additives [1]. Despite high self-sufficiency, China’s growing demand for animal-based foods has intensified competition for resources between humans and livestock. Therefore, it is imperative to enhance the utilization efficiency of poultry feed resources.

To address this, three key strategies can be implemented to enhance production efficiency and reduce feed consumption: 1) precise analysis of feed nutrients; 2) exploration and utilization of agricultural by-products as feed ingredients; 3) research on the digestive and physiological characteristics of Chinese poultry breeds.

### Feed analysis and database construction

The database of nutritional values for feed raw materials serves as the foundation for precise feed formulation. To accurately assess the nutritional value of feed, our team developed a five-dimensional evaluation system, which includes comprehensive nutritional analysis, pure nutritional analysis, anti-nutritional factors, mycotoxins, and physicochemical properties. Drawing from the feed databases of the United States and Brazil, China established its own National Feed Database, based on domestic feed resources.

Moreover, researchers are actively exploring new feed ingredients, aiming to reduce reliance on conventional feed ingredients. The exploration of new feed raw materials focuses on three areas: crop by-products, food processing by-products, and engineered bacteria for synthesizing glucose and amino acids. Crop by-products typically refer to underutilized plant stems and leaves, such as sweet potato residues, maple leaves, and others. For example, Acer truncatum leaf extract, a unique Chinese prebiotic, reduced lipopolysaccharide-induced gut damage in broilers by modulating immune and antioxidant functions [24]. Food processing by-products refer to animal-derived materials produced during processing, including feathers, fat, eggshells, and others. These by-products can be incorporated into feed based on their nutritional value. Highly efficient engineered bacterial systems use low-cost, readily available raw materials to synthesize target substances. Glucose was synthesized by cyanobacteria using carbon dioxide as the substrate [25].

### Nutritional and metabolic disorders

The feed efficiency of high-performance poultry in China has reached international leading levels. However, these poultry exhibit nutritional and metabolic disorders, leading to production efficiency falling below global standards. In broiler and layer farms, the incidence of conditions such as woody meat, glandular gastritis, ventriculitis, pulmonary inflammation, and necrotizing enteritis remains high. These disorders arise because rapid breeding has resulted in feed formulations that fail to meet the nutritional requirements for healthy growth. For example, broilers are more sensitive to amino acids due to the increased meat yield from year-to-year breeding. Additionally, local breeds lack comprehensive nutritional and feeding standards, leading to higher production costs.

### Intestinal health

In 2020, the Chinese government banned the use of antibiotics in livestock feed, which has led to significant challenges in maintaining intestinal health in poultry. Feed designed based on the physiological characteristics of poultry intestines can enhance intestinal health and improve production efficiency. However, significant gaps remain in the fundamental research on poultry intestinal function, limiting the resolution of intestinal health issues and hindering further improvements in production efficiency.

To address this issue, our team come up with the concept of “nutrition power”, which is defined as the notion that nutrition is a dynamic process, with each part of the gut functioning as both distinct and interconnected. Meanwhile, our team come up with the “five-ring gold standard” for evaluating intestinal health, encompassing five dimensions: (1) strong digestion and absorption, (2) complete physical barrier, (3) specific chemical barrier, (4) stable microbiota, and (5) moderate mucosal immunity. The concept of “nutrition power” and the gold standard of the intestinal five rings provide researchers with valuable tools to better understand and analyze the digestive physiological characteristics of poultry.

#### Strong digestion and absorption

The digestion and absorption efficiency of feed nutrients is the most intuitive index to evaluate the digestion and absorption function of livestock and poultry, which can be determined by digestion test and metabolism test [26]. In addition, the digestion and absorption function of livestock and poultry can also be evaluated by measuring key factors in the process of digestion and absorption, such as digestive enzymes and transporters [27]. For example, our research examined the developmental characteristics of digestive and absorptive capacity in Arbor Acres broilers [28].

#### Complete physical barriers

Physical barriers ensure the digestion and absorption of nutrients in the alimentary tract, maintain the permeability of the alimentary tract, and prevent harmful substances or pathogenic microorganisms from entering the body. Physical barriers, including villi, crypts, and tight junctions, maintain gut permeability and nutrient absorption while preventing pathogen entry [29]. Our studies found that accessible fiber, acting as a microbial substrate, regulates the mucosal barrier through short-chain fatty acids and bile acids [30]. Additionally, Chinese herbs, such as astragalus polysaccharide, enhance barrier function by increasing the abundance of Parabacteroides in the gut [31].

#### Specific chemical barriers

The specific chemical barrier of the digestive tract not only maintains the homeostasis of flora and prevents harmful microorganisms from contacting the intestinal epithelium, but also plays a signal regulation role through the specific binding of microbial metabolites to specific sites to maintain the health of the gut and the host. The digestive tract chemical barrier mainly includes the internal acid-base environment, volatile fatty acids, digestive enzymes, bile acids and other epithelial cell secretions or microbial metabolites [32]. For example, injecting Lactobacillus plantarum (isolated from Chinese sauerkraut) into Arbor Acres broilers embryos promoted beneficial gut microbiota and short-chain fatty acid production, improving poultry performance [33].

#### Stable microbiota

Stable gastrointestinal microbiota is of great significance to inhibit the colonization of pathogens in the intestine, improve livestock performance and feed use efficiency [34].

Probiotics and prebiotics help establish healthy gut microbiota. Our lab found that oregano extract supplementation improved broiler productivity by improving beneficial microorganisms growth [35]. Similarly, folic acid supplementation influenced microbial regulation of lipid metabolism, enhancing poultry performance [36,37]. Besides, recent studies have focused on the establishment and differentiation of microbiota-host-gut types, and microbial colonization is affected by diet, age, and gut location, as well as their interactions [38]. Among them, the temporal and spatial abundance of some specific microbial taxa is related to feed efficiency traits of livestock and poultry [39,40], which can enable the host to effectively use dietary proteins and carbohydrates, thereby improving feed use efficiency [41]. Regulation of microbial-host interactions may be considered in future work as a nutritional strategy to improve feed efficiency in livestock and poultry.

#### Moderate mucosal immunity

The mucosal immune system is the site where the intestine contacts foreign antigens, initially forms immune responses, and performs local non-specific immune functions. Secretory immunoglobulin A (sIgA) plays an important role in intestinal mucosal immunity, including immune rejection, antigen presentation, and interaction with the intestinal symbiont [42,43]. In addition, the expression of intestinal cytokines is also an important indicator. In particular, the levels of pro-inflammatory factors TNF-α, IL -1β, IL-6, IFN-γ and anti-inflammatory factor IL-10 were detected.

## SMART POULTRY FARMING

In recent years, China’s poultry farming model has undergone a significant transformation, shifting from traditional small-scale family farming to large-scale and intelligent farming systems. The traditional methods often fail to efficiently monitor and adjust environmental conditions and animal physiological parameters, which constrains improvements in poultry production efficiency. The intelligent poultry farm management platform integrates environmental control systems that monitor real-time environmental parameters and physiological data of poultry health to manage poultry houses efficiently.

However, the implementation of smart poultry farm management platforms still faces three key challenges: 1) Penetration remains relatively low; 2) Smart equipment require further optimization, and 3) Poultry health indicators need to be expanded and their accuracy enhanced. Our team conducts comprehensive research on smart poultry farming, covering all aspects, including farm design, management in intelligent poultry farming, precision feeding, intelligent health monitoring, data analysis platform, environmental monitoring, waste utilization and resource recycling.

### Farm design and management in intelligent poultry farming

The design of farms for intelligent poultry farming should fully consider the living environment of the animals and production efficiency. A reasonable layout of the breeding area, feeding area, and waste treatment area is essential to ensure an efficient and sustainable production process. Additionally, the design should integrate the installation locations of smart devices to facilitate data collection and environmental monitoring [44]. Implement an intelligent management system to oversee the entire poultry farming process through a centralized control platform. This system should integrate environmental data, health monitoring, feed management, and other relevant information, providing real-time feedback to assist farmers in making informed and timely decisions to optimize production efficiency and poultry welfare [45].

In intensive agriculture, the application of intelligent facilities is particularly crucial. High-density farming can be achieved through centralized monitoring and automated equipment, thereby increasing production efficiency per unit area. This model is suitable for large-scale operations and offers significant economic benefits [46]. Both large-scale and family farms benefit from intelligent facilities, as they enable efficient resource management and optimize production across various farming scales. For family farming, intelligent facilities are equally important. Small-scale farmers can use smart devices to monitor the health and growth conditions of their poultry, enabling precise management and improving production efficiency. This model not only contributes to the economic development of families but also supports the diversification of rural economies.

### Precision feeding

In modern poultry farming, automated equipment such as intelligent feeders and manure removal systems are becoming increasingly prevalent. These devices help reduce manual labor, enhance operational efficiency, and ensure that poultry receive the necessary feed and water on time [47]. By integrating Internet of Things (IoT) technology with automated equipment, farmers can achieve precise control over both feeding and waste management, improving overall farm efficiency. By utilizing IoT technology, farmers can implement precision feeding by automatically adjusting feed formulations and quantities based on each poultry’s growth stage and health status [48]. This method not only optimizes feed utilization but also minimizes feed waste.

### Intelligent health monitoring in poultry

Poultry farming faces considerable challenges under extreme climatic conditions [49]. Intelligent temperature control systems help address these issues by automatically adjusting indoor temperature and humidity based on real-time meteorological data, thereby ensuring optimal conditions for poultry health and minimizing the risk of heat or cold stress. The environmental monitoring system employs sensors to track real-time indicators such as temperature, humidity, and ammonia concentration, ensuring that the farming environment remains optimal. The system can automatically adjust ventilation, heating, and other equipment to maintain ideal conditions for poultry growth [46]. This technology not only enhances poultry growth performance but also reduces disease risks by maintaining a stable environment.

In addition to temperature control, real-time health monitoring systems play a crucial role in intelligent poultry farming. With the use of wearable devices and monitoring cameras, farmers can continuously track poultry activity, feeding habits, and drinking behavior. Data analysis enables the early detection of health issues, such as reduced appetite or decreased activity, allowing for timely intervention. This real-time monitoring system not only supports poultry growth but also effectively reduces the risk of disease transmission [44]. Together, intelligent temperature and health monitoring systems form an integrated approach to maintaining poultry welfare and productivity, especially under challenging environmental conditions.

### Data analysis platform

Big data technology can be leveraged to conduct comprehensive analyses of data generated throughout the poultry farming process, providing deeper insights and enabling informed decision-making to optimize production efficiency and resource management. This includes feed consumption, poultry growth rates, and health conditions [50]. By analyzing this data in real time, farmers can identify trends and make proactive adjustments to optimize poultry management. Data-driven decision support enhances production efficiency by facilitating informed management decisions [51].

### Waste utilization and resource recycling

Intelligent poultry farming not only focuses on production efficiency but also emphasizes effective waste management and resource utilization. Using advanced treatment technologies, manure generated on the farm can be transformed into organic fertilizer for field application, thereby achieving resource recycling. This approach reduces environmental pollution while providing high-quality soil enhancement materials for agriculture [52]. By integrating waste recycling systems with intelligent facilities, farms can not only improve production efficiency but also contribute to environmental sustainability.

As technology continues to advance, intelligent farming will play an increasingly important role in both intensive agriculture and family farming. In the future, intelligent poultry farming is expected to provide strong support for achieving sustainable agriculture and contribute more to global food security.

## POULTRY-CROP INTEGRATING SYSTEMS

**Figure 2:**
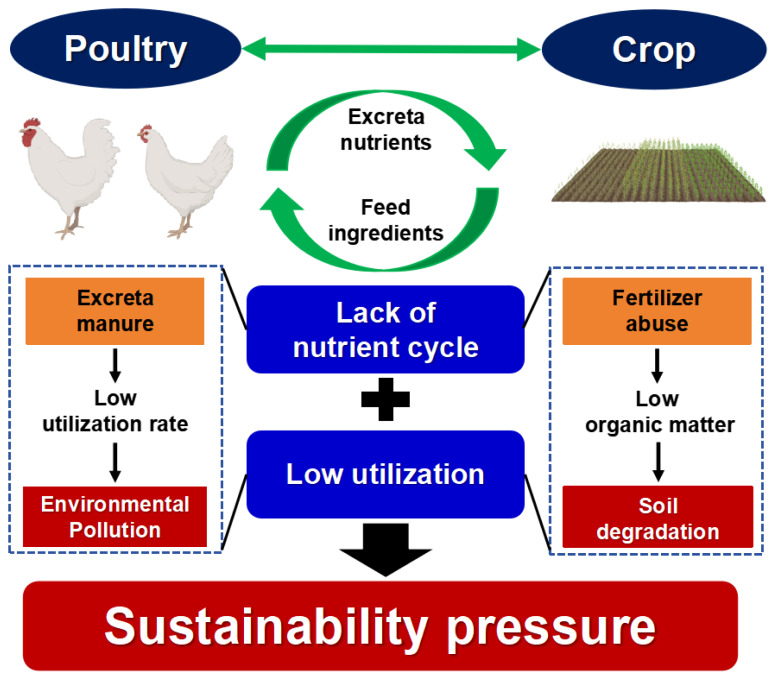
Poultry-Crop Integrating systems. The separation of poultry industry and crop industry leads to low nutrient utilization efficiency, which affects the sustainable development of poultry industry. On the one hand, the low manure utilization efficiency of poultry industry leads to environmental pollution, and on the other hand, the soil compaction caused by excessive dependence on fertilizer of crop industry. Poultry manure and crop cultivation should be closely integrated to effectively recycle nutrients and foster the sustainable development of the poultry industry.

With the growth of the global population and the increasing demand for food, sustainable agricultural development is confronted with numerous challenges (Figure 2). The significant amount of waste produced by poultry farming is underutilized, contributing to non-point source pollution and nutrient waste, while crop production remains heavily reliant on chemical fertilizers, resulting in a decline in soil organic matter and land degradation [53]. This inefficient model results in insufficient resource interaction, undermining the sustainability of agriculture [54]. Therefore, integration crop and poultry farming to promote the efficient recycling of nutrient resources is imperative [55].

Crop production and poultry farming form material exchange cycles, collectively referred to as Poultry-Crop Interacting systems. Currently, some Chinese poultry enterprises use poultry manure as fertilizer to enhance material utilization efficiency. However, the application ratio and overall efficiency remain low. Poultry-Crop Interacting systems face four key challenges: (1) The separation of crop production and poultry farming [53,56]; (2) The construction of a fully integrated circular system linking crop production and poultry breeding remains incomplete; (3) A standardized cycle parameter system for the system has not yet been established; (4) There is significant potential to further improve material utilization efficiency across all aspects of the system.

### Basic concept of Poultry-Crop Integrating systems

Integrated crop-poultry systems, also known as “ecological agricultural cycles” or “agricultural circular economies”, are a production model that organically combines the planting and breeding stages in agriculture [57]. The fundamental principle behind this approach is to convert waste generated during poultry farming, such as poultry manure, into organic fertilizers for crops, while simultaneously using by-products from crop production, such as crop straw and weeds, as poultry feed. This system promotes the efficient recycling of resources, reduces external inputs, and minimizes environmental pollution [58,59].

In poultry farming, poultry manure is rich in essential nutrients such as nitrogen, phosphorus, and potassium, making it an excellent source of organic fertilizer [60]. However, if this waste is directly discharged, it can cause environmental pollution and lead to the wastage of valuable resources [61]. Therefore, integrating poultry farming with crop production in a unified system offers an effectively solution to this issue, efficiently utilizing manure while also absorbing excess nutrients from the soil through crop cultivation. This approach helps prevent soil fertility imbalances and reduces the risk of further environmental pollution [62].

### Specific technologies in Poultry-Crop Integrating systems

The treatment and utilization of manure are critical processes in poultry farming. Through methods such as composting and anaerobic fermentation, poultry manure can be converted into high-quality organic fertilizers [63]. Specific technologies include: (1) Composting: this method involves mixing poultry manure with plant residues, and managing humidity and ventilation to encourage microbial degradation, ultimately producing high-quality organic fertilizer. Studies show that composting not only reduces dependence on chemical fertilizers but also improves soil fertility and structure [64]. (2) Anaerobic fermentation: transforming manure into biogas and organic fertilizers in an oxygen-free environment. Biogas can be used for electricity generation or heating, reducing fossil fuel use, while the remaining organic fertilizer can be applied to farmland to promote crop growth [65].

Ecological breeding emphasizes leveraging natural processes to maintain ecological balance and improve farm sustainability. For instance, introducing animals with different feeding behaviors, such as ducks or geese, into chicken farms can not only reduce disease transmission but also enhance resource utilization efficiency, contributing to a more diversified and resilient farm ecosystem. This method increases biodiversity within the farm, which in turn strengthens overall production performance [66].

Similarly, in waterfowl farming, resource recycling-particularly of water is essential for sustainable operations [67]. By incorporating a recirculating aquaculture system, wastewater from waterfowl farms can be purified and repurposed for irrigating farmland, reducing water wastage and improving resource efficiency. Additionally, filtration and recycling technologies not only conserve water but also create a healthier growth environment for waterfowl. Together, these ecological breeding practices demonstrate how sustainable methods can enhance production while supporting environmental conservation.

### The Poultry-Crop Integrating systems in China

Our research team founded the Institute of Poultry-Crop Integrating Systems, dedicated to advancing several core areas: feed preparation, poultry farming, product processing, manure treatment and crop cultivation.

#### Feed preparation

Poultry feed represents a significant portion of farming costs, with traditional feed sources primarily relying on external procurement. However, in Poultry-Crop Integrating systems, by-products from the planting stage, such as straw, corn cobs, and soybean stalks, can be easily processed and used as poultry feed [68]. This approach not only reduces farming costs but also alleviates the burden of agricultural waste disposal [69]. For example, corn straw can be crushed and mixed with other feed ingredients to serve as coarse feed for chickens and ducks; while rice bran can be incorporated into poultry diets. This method effectively utilizes waste from the planting stage, creating a closed-loop resource cycle.

#### Poultry farming and product processing

Poultry farming in China is categorized into intensive farming and ecological farming. Intensive farming is efficient for collecting poultry waste, making centralized disposal easier compared to ecological farming. Currently, intensive poultry enterprises in China have begun returning treated poultry manure to fields. However, the proportion of manure being recycled back into fields and the efficiency of this process remain low.

Ecological free-range farming is a poultry farming method that integrates the concept of Poultry-Crop Integrating Systems. The “rice-duck co-culture” model is a successful example promoted in rural China [70]. In this model, poultry are allowed to roam freely in planting areas, where they can forage on insects, weeds, and crop residues while their manure is directly deposited in the fields, serving as natural fertilizer [71]. For example, in orchards, chickens peck at insect eggs and weeds on the ground, thereby reducing the need for pesticide, while their manure enriches the soil. In rice paddies, ducks can eat pests and weeds, decreasing the reliance on pesticides and herbicides, while their droppings act as fertilizer, promoting rice growth. This “pastoral” free-range model not only reduces feed consumption but also promotes soil fertility cycling [72]. This approach reduces the reliance on chemical fertilizers and pesticides, while significantly enhancing both crop yield and quality [73].

The processing of poultry products generates various by-products, such as feathers, fat, and blood, which can be effectively utilized as feed raw materials to enhance the efficiency of resource utilization. However, the current reuse of these by-products remains insufficient. The primary challenges include a lack of recognition of their value and the absence of specialized recycling channels.

#### Manure treatment and crop cultivation

Poultry manure is an efficient organic fertilizer that, when proper treated, can be directly returned to the fields. This enhances soil structure, increases organic matter content, and improves soil fertility. The nutrient content, particularly nitrogen, in poultry manure significantly promotes crop growth [74]. However, applying untreated poultry manure directly can lead to environmental pollution and the spread of pathogens, necessitating treatment through composting or fermentation to produce harmless organic fertilizer. In practice, farms typically send collected poultry manure to composting facilities for fermentation. High-temperature fermentation kills pathogens and weed seeds while decomposing organic matter, forming stable humus. This treated organic fertilizer not only provides nutrients for crops but also improves soil structure and increases the soil’s ability to retain water and nutrients [75].

### Future development directions

Poultry-Crop Interacting systems present broad application potential in modern poultry farming, especially to meet the increasing demands for sustainable agricultural practices. Future development should prioritize technological innovation to enhance efficiency and environmental benefits. This includes advancing research in areas such as manure management, waste utilization, and ecological breeding technologies, which collectively improve production efficiency and support eco-friendly farming practices.

In addition to technological advancement, strong policy support from the government is crucial for promoting integrated systems. Offering targeted subsidies and technical guidance will encourage farmers to adopt these sustainable practices more widely. Alongside policy initiatives, comprehensive education and training programs are essential to increase farmers’ understanding of integrated systems and improve their practical skills, allowing them to implement effective crop-poultry integration in real production contexts.

Poultry-Crop Interacting systems are increasingly recognized as a promising solution for addressing the challenges of crop and poultry production when managed separately. By employing strategies such as cross-medium integration, source reduction, efficiency enhancement, and effective resource coupling, these systems enable efficient nutrient cycling, improve soil fertility, and optimize food production efficiency. With continuous technological advancement and strong policy support, Poultry-Crop Interacting systems are expected to become a cornerstone for achieving sustainable agriculture.

## CONCLUSION

China’s poultry industry is vast in scale and diverse in breed, presenting numerous opportunities as well as challenges. In genetic breeding, researchers have leveraged China’s rich poultry genetic resources to bred high-performance breeds characterized by exceptional production efficiency, nutritional value, and resilience to environmental stress. In the field of feed nutrition, a National Feed Database and a Five-Dimensional Feed Evaluation System were developed to address the limitations in feed resource utilization and nutritional value evaluation in China. Guided by the principles of “nutrition power” and the “five-ring gold standard”, researchers systematically analyzed the nutritional metabolism characteristics of Chinese poultry. The implementation of intelligent farming systems enhanced the effectiveness of genetic and nutritional improvements by enabling real-time monitoring and automated environmental adjustments. Furthermore, the Poultry-Crop Interacting systems promote resource-efficient recycling and environmental sustainability by creating integrated cycles for poultry production and crop utilization. In conclusion, China’s poultry industry is progressing toward a sustainable, intelligent, and efficient future. Achieving this vision will require interdisciplinary collaboration across genetics, nutritional science, and smart agriculture.
